# Sodium Selenite Regulates the Proliferation and Apoptosis of Gastric Cancer Cells by Suppressing the Expression of LncRNA HOXB-AS1

**DOI:** 10.1155/2022/6356583

**Published:** 2022-11-21

**Authors:** Hongsheng Jiang, Lingbo Hu, Quanfeng Wu, Bitao Zhang, Jianhua Sun, Xiaoying Li

**Affiliations:** ^1^Department of Gastrointestinal Surgery, The Central Hospital of Enshi Tujia and Miao Autonomous Prefecture, Enshi 445000, Hubei, China; ^2^Hubei Selenium and Human Health Institute, The Central Hospital of Enshi Tujia and Miao Autonomous Prefecture, Enshi 445000, Hubei, China; ^3^Department of Health Management Center, The Central Hospital of Enshi Tujia and Miao Autonomous Prefecture, Enshi 445000, Hubei, China

## Abstract

Gastric carcinoma has a high incidence, accounting for approximately 6% of all cancers worldwide. The in vivo antitumor effect of sodium selenite on gastric carcinoma has been demonstrated. This study therefore aimed to further explore its targets in gastric cancer in vitro and elucidate its mechanism of action. The effects of inorganic sodium selenite (Na_2_SeO_3_) on apoptosis, proliferation, and invasion of gastric cancer cells were investigated, and the interaction between Na_2_SeO_3_ and expression of long noncoding RNA homeobox B cluster antisense RNA 1 (HOXB-AS1) was investigated to elucidate the specific mechanism of action of selenium on gastric cancer cell proliferation through regulation of HOXB-AS1. Na_2_SeO_3_ downregulated the expression of HOXB-AS1 in the human gastric cancer (HGC) cell lines, HGC-27, NCI-N87, and KATO III cells, while inhibiting their proliferation and invasion and inducing apoptosis. The upregulation of HOXB-AS1 produced the opposite results. Na_2_SeO_3_ was used to stimulate HGC-27 cells, which caused HOXB-AS1 overexpression. The cell counting kit-8 (CCK-8) assay revealed a decrease in cell proliferation, while western blotting, flow cytometry, and transwell migration assays showed the expression of apoptosis-related (Bad, Bcl-2, and cleaved-caspase-3) and invasion-related (MMP2, E-cadherin, and N-cadherin) proteins, indicating increased apoptosis and decreased invasion. We therefore conclude that Na_2_SeO_3_ inhibits the malignant progression of gastric cancer by downregulating the expression of HOXB-AS1 and thus could be used as a potential drug for its treatment.

## 1. Introduction

Gastric carcinoma ranks fifth and third in terms of the incidence and mortality rates of malignant tumors worldwide [1]. Although radical surgery is the preferred treatment modality for gastric cancer, standard treatment also involves perioperative chemotherapy [2, 3]. Most chemotherapeutic agents are toxic and exert adverse effects. In addition, 50% of patients with advanced gastric cancer show varying degrees of recurrence after receiving standard adjuvant therapy [4].

Selenium is an essential trace element that plays an important role in various physiological functions of the human body, including health maintenance and disease prevention [5]. Selenium occurs in organic (selenomethionine, selenocysteine, and *γ*-glutamyl-Se-methyl selenocysteine) and inorganic (sodium selenate and sodium selenite) forms [6, 7]. One study reported that people who consumed a selenium-rich diet had a lower risk of cancer [8]. Selenium can improve immunity and reduce DNA damage and oxidative stress [9]. The results of scientific research in the last century have shown that sodium selenite, sodium selenate, selenomethionine, selenium methylselenocysteine, and methylselenic acid all exert obvious antitumor effects [10, 11].

Long noncoding RNAs (lncRNAs) are RNA molecules that are longer than 200 nucleotides but have no protein coding ability [12]. In recent years, substantial evidence has suggested that lncRNAs play an important role in cancer by participating in proliferation, migration, apoptosis, and other tumor cell activities [13, 14]. Yan et al. found that the overexpression of lncRNA-MUF could activate the Wnt/*β*-catenin signaling pathway and promote epithelial mesenchymal transition by forming a complex with membrane-binding protein A2, thus promoting the development of liver cancer [15]. lncRNA H19 promotes cell proliferation by competitively binding to miR-200a and derepressing *β*-catenin expression in colorectal cancer [16]. Selenium can further regulate the transcription of genes and affect lncRNA expression [17]. HOXB-AS1, a newly discovered lncRNA, and a similar encoded short peptide, HOXB-AS3, have been reported to inhibit the proliferation and metabolism of colon cancer cells [18, 19]. However, the mechanism underlying the interaction between selenium and HOXB-AS1 in gastric cancer remains unclear.

In this study, the effects of Na_2_SeO_3_ and HOXB-AS1 on apoptosis, proliferation, and invasion of gastric cancer cells as well as the interaction between selenium and HOXB-AS1 were investigated. Our results suggest that Na_2_SeO_3_ inhibits gastric cancer pathogenesis by downregulating HOXB-AS1 expression.

## 2. Materials and Methods

### 2.1. Cell Culture

Human gastric cancer (HGC) cells (HGC-27, NCI-N87, and KATO III) and a human gastric epithelial cell line (GES-1) were obtained from the Shanghai Cell Bank of the Chinese Academy of Science and cultured at 37°C in 5% CO_2_. The cells were passaged at a ratio of 1:2–4 after reaching a confluency of >90%. HGC cells were grown in the RPMI-1640 medium (Hyclone) supplemented with 10% fetal bovine serum (FBS). GES-1 cells were cultured in Dulbecco's modified Eagle's medium (DMEM) (Hyclone) supplemented with 10% FBS.

### 2.2. Cell Transfection

HGC-27 cells were transfected with si-HOXB-AS1/si-NC or ov-HOXB-AS1/ov-NC using Lipofectamine 2000 (Invitrogen). Three different si-HOXB-AS1 sequences were inserted into the plasmid vector: siRNA-1(5′-GTGGAAGAAACCGATAATT-3′),siRNA-2(5′-CTCCGTTTCTCCAGAAAAG-3′), and siRNA-3(5′-CCAGCGAAATTACAGGGAA-3′), all synthesized by Guangzhou Ruibo Biotechnology Co., Ltd. After 48 h of transfection, the cells were collected and used to analyze transfection efficiency by quantitative reverse transcription-polymerase chain reaction (RT-PCR).

### 2.3. Cell Treatment

HGC and GES-1 cells were treated with different concentrations of Na_2_SeO_3_ (0, 15, 30, and 60 *μ*M) for 48 h. Cells were randomly divided into five groups: control (no treatment, CON), Na_2_SeO_3_ (treated with 15 *μ*M Na_2_SeO_3_ for 48 h), ov-HOXB-AS1 (transfected with HOXB-AS1), ov-HOXB-AS1 + Na_2_SeO_3_ (transfected with HOXB-AS1 and treated with 15 *μ*M Na_2_SeO_3_ for 48 h), and ov-NC + Na_2_SeO_3_ (transfected with ov-NC and treated with 15 *μ*M Na_2_SeO_3_ for 48 h).

### 2.4. CCK-8 Assay

The CCK-8 (Solarbio) assay was performed to evaluate cell viability. Cells were seeded in 96-well plates (3 × 10^3^ cells/well) and cultured overnight at 37°C in 5% CO_2_. After treatment, 10 *μ*L of the CCK-8 solution was added to each well and incubated for 4 h. The absorbance of each well was measured at 450 nm with a microplate reader.

### 2.5. Flow Cytometry

Apoptosis was investigated using flow cytometry. A cell suspension containing 1 × 10^6^ cells was washed and centrifuged with ice-coldphosphate-buffered saline at 800 × g for 5 min. The cells were then stained with 10 *μ*L annexin V-FITC and 10 *μ*L propidium iodide for 20 min in the dark before being subjected to flow cytometry.

### 2.6. Western Blot

The total protein in the supernatant was quantified using a BCA protein concentration assay kit (Solarbio). Proteins (20 *μ*g/lane) were separated using 10% sodium dodecyl sulfate-polyacrylamide gel electrophoresis and transferred onto polyvinylidene fluoride membranes. After blocking with 5% skimmed milk overnight at 4°C, the membranes were incubated with specific primary antibodies against Bad (Bioswamp, PAB32756, 1 : 1000), B-cell lymphoma (Bcl)-2 (Bioswamp, PAB33482, 1 : 1000), cleaved-caspase-3 (Abcam, ab2302, 1 : 1000), MMP2 (Bioswamp, PAB34434, 1 : 1000), E-cadherin (Bioswamp, PAB33542, 1 : 1000), N-cadherin (Bioswamp, PAB30131, 1 : 1000), and GAPDH (Bioswamp, PAB36269, 1 : 1000) for 1 h at 25 ± 2°C. Next, the membranes were incubated with a goat anti-rabbit IgG secondary antibody (Bioswamp, SAB43714, 1 : 20000) at 25°C ± 2°C for 1 h and visualized using an enhanced chemiluminescence system.

### 2.7. Transwell Migration Assay

HGC-27 cells were suspended in a serum-free medium with 1% FBS and seeded (1 × 10^5^ cells/well) in the upper chamber of the transwell; 0.75 mL of the serum-free medium with 10% FBS was then added to the lower chamber. After 48 h of coincubation at 37°C, the HGC-27 cells on the bottom surface of the lower chamber were fixed with 4% formaldehyde for 20 min at room temperature and stained with 0.5% crystal violet for 30 min. Cells that did not migrate to the lower chamber were counted under an optical microscope (200× magnification).

### 2.8. RT-PCR

Total RNA was extracted from the collected cells using TRIzol reagent (Ambion) and reverse-transcribed into cDNA using the M-MuLV kit (TaKaRa, Dalian, China). cDNA was used as a template for qPCR amplification. The PCR conditions were as follows: predenaturation at 95°C for 3 min, denaturation at 95°C for 5 s, annealing at 56°C for 10 s, and extension at 72°C for 25 s. A total of 40 cycles were performed, with glyceraldehyde 3-phosphate dehydrogenase (GAPDH) used as an internal reference for intersample calibration. The primer sequences were as follows: HOXB-AS1-F, 5′-CTACACCAGTGCCTCACA-3′ HOXB-AS1-R, 5′-CTTCCACGAAACCTAAACGAPDH-F, 5′-GGGAAACTGTGGCGTGAT-3′; GAPDH-R, and 5′-GAGTGGGTGTCGCTGTTGA-3′. The expression of HOXB-AS1 was calculated using the 2^−ΔΔCt^ method.

### 2.9. Statistical Analysis

Data are presented as the mean ± standard deviation. One-way ANOVA was used to compare the differences between the groups using SPSS 21.0. Statistical significance was set at *P* ≤ 0.05.

## 3. Results

### 3.1. Effect of Na_2_SeO_3_ on Apoptosis, Migration, and Proliferation of HGC-27 Cells

**Figure 1:**
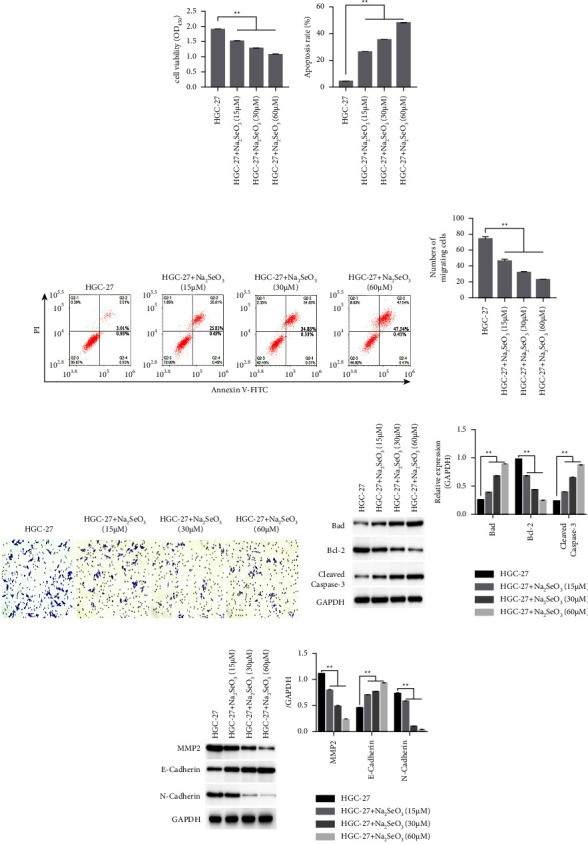
Effect of Na_2_SeO_3_ on proliferation, apoptosis and migration of HGC-27 cells. (a) The viability of HGC-27 cells was determined using a CCK-8 assay kit. (b, c) The level of apoptosis of HGC-27 cells was assessed using FCM. (d, e) Migration ability of HGC-27 cells was detected by a transwell assay. (f) Relative expression of apoptosis-related proteins was detected using western blotting. (g) Relative expression of migration-related proteins was detected by western blotting. The data are expressed as the mean ± SD (*n* = 3). ^*∗∗*^represents *P* < 0.01. Data are expressed as the mean ± standard deviation [SD] (*n* = 3), ^*∗∗*^represents *P* < 0.01 and ^*∗*^represents *P* < 0.01.

The viability (Figure 1(a)) and migration ability (Figures 1(d) and 1(e)) of HGC-27 cells were significantly decreased in groups treated with the three different doses of Na_2_SeO_3_ (15, 30, and 60 *μ*M) compared to the controls (*P* < 0.01). Apoptosis was significantly increased in HGC-27 cells treated with Na_2_SeO_3_ (*P* < 0.01, Figures 1(b) and 1(c)) compared to the controls. In addition, apoptosis-related and migration-related protein expression (Figures 1(f) and 1(g)) was detected in HGC-27 cells with or without drug treatment. The protein expression of Bad, cleaved-caspase-3, and E-cadherin was significantly upregulated in groups treated with Na_2_SeO_3_ compared to that in the control group (*P* < 0.01). In contrast, the expression of Bcl-2, MMP2, and N-cadherin proteins was significantly downregulated compared to the control group (*P* < 0.01, Figures 1(d) and 1(e)). In all cases, the effects of Na_2_SeO_3_ were dose-dependent.

### 3.2. Effect of Na_2_SeO_3_ on HOXB-AS1 mRNA Expression in Human Gastric Cancer Cells (HGC-27, NCI-N87, and KATO III) and GES-1 Cells

**Figure 2:**
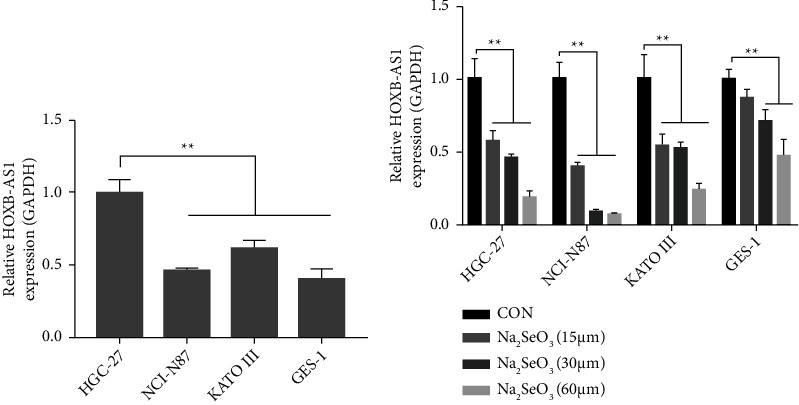
Effect of Na_2_SeO_3_ on HOXB-AS1 mRNA expression in human gastric cancer cells (HGC-27, NCI-N87 and KATO III) and GES-1 cells. (a) Relative HOXB-AS1 expression in human gastric cancer cells (HGC-27, NCI-N87 and KATO III) and GES-1 cells, as determined by qRT-PCR. (b) Relative HOXB-AS1 expression in human gastric cancer cells (HGC-27, NCI-N87 and KATO III) and GES-1 cells after treatment with different concentrations of Na_2_SeO_3_ (0, 15, 30 and 60 *μ*M) for 48 h, as determined by RT-PCR. Data are expressed as the mean ± standard deviation [SD] (*n* = 3), ^*∗∗*^represents *P* < 0.01 and ^*∗*^represents *P* < 0.01.

We detected the expression of HOXB-AS1 mRNA in the HGC-27, NCI-N87, and KATO III cells, as well as in GES-1 cells, using qRT-PCR. The mRNA expression of HOXB-AS1 in HGC-27 cells was significantly higher than that in NCI-N87, Kato III, and GES-1 cells (*P* < 0.01; Figure 2(a)). Next, the four cell types were treated with different concentrations of Na_2_SeO_3_ (0, 15, 30, and 60 *μ*M) for 48 h. Compared to controls, the expression of HOXB-AS1 mRNA was significantly downregulated in all cells treated with Na_2_SeO_3_ (*P* < 0.01, Figure 2(b)). HGC-27 cells were selected as the cell model for subsequent experiments.

### 3.3. Effect of HOXB-AS1 on Apoptosis, Migration, and Proliferation of HGC-27 Cells

**Figure 3:**
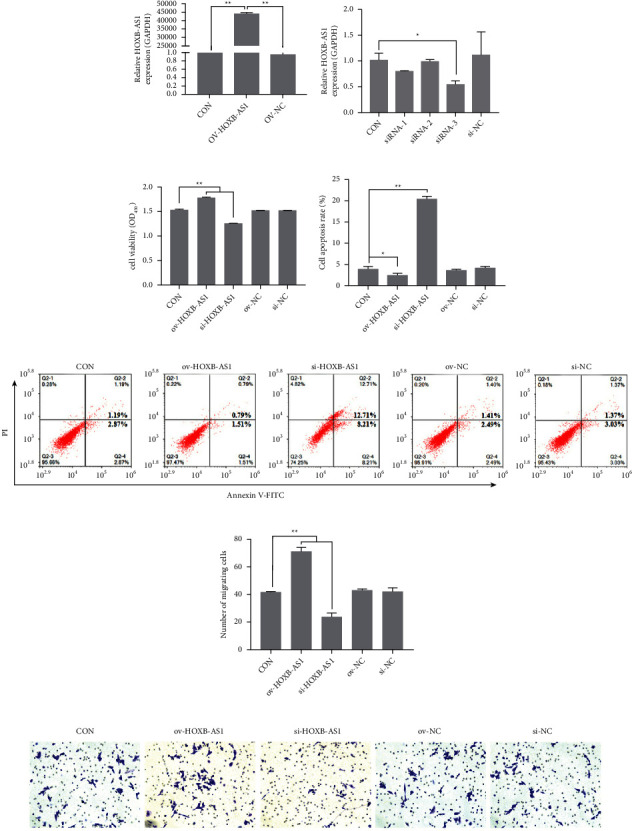
Effect of HOXB-AS1 on proliferation, apoptosis and migration of HGC-27 cells. (a) Relative expression of HOXB-AS1 in Huh-7 cells after transfection with ov-HOXB-AS1 or the corresponding negative control (ov-NC), as determined by RT-PCR. (b) Relative expression of HOXB-AS1 in Huh-7 cells after transfection with siRNA-1, siRNA-2, siRNA-3, or the corresponding negative control (si-NC), as determined by RT-PCR. (c) The viability of HGC-27 cells was determined using a CCK-8 kit. (d, e) The level of apoptosis of HGC-27 cells was assessed using FAM. (f, g) The migration ability of HGC-27 cells was detected by a transwell assay. Data are expressed as the mean ± standard deviation [SD] (*n* = 3), ^*∗∗*^represents *P* < 0.01 and ^*∗*^represents *P* < 0.01.

After transfection, the expression of HOXB-AS1 was measured using qRT-PCR. Cells transfected with ov-HOXB-AS1 showed significantly higher expression of HOXB-AS1 than the CON group and the corresponding negative control (ov-NC) group (*P* < 0.01, Figure 3(a)). In contrast, cells transfected with siRNA-3 showed significantly lower expression of HOXB-AS1 than the CON group (*P* < 0.01, Figure 3(b)). There were no differences in the expression of HOXB-AS1 between the siRNA-1, siRNA-2, si-NC, and CON groups. Compared to the CON group, the viability (Figure 3(c)) and migration ability (Figures 3(f) and 3(g)) of HGC-27 cells had significantly increased in the ov-HOXB-AS1 group (*P* < 0.01) but significantly decreased in the si-HOXB-AS1 group, transfected with siRNA-3 (*P* < 0.01). The apoptosis of HGC-27 cells was determined using flow cytometry (Figures 3(d) and 3(e)). Compared to the control group, apoptosis of HGC-27 cells was increased in the si-HOXB-AS1 group and significantly decreased in the ov-HOXB-AS1 group. There were no differences in viability, migration ability, and apoptosis among the siRNA-1, siRNA-2, si-NC, and CON groups.

### 3.4. Effect of Na_2_SeO_3_ on HOXB-AS1-Influenced HGC-27 Cells

**Figure 4:**
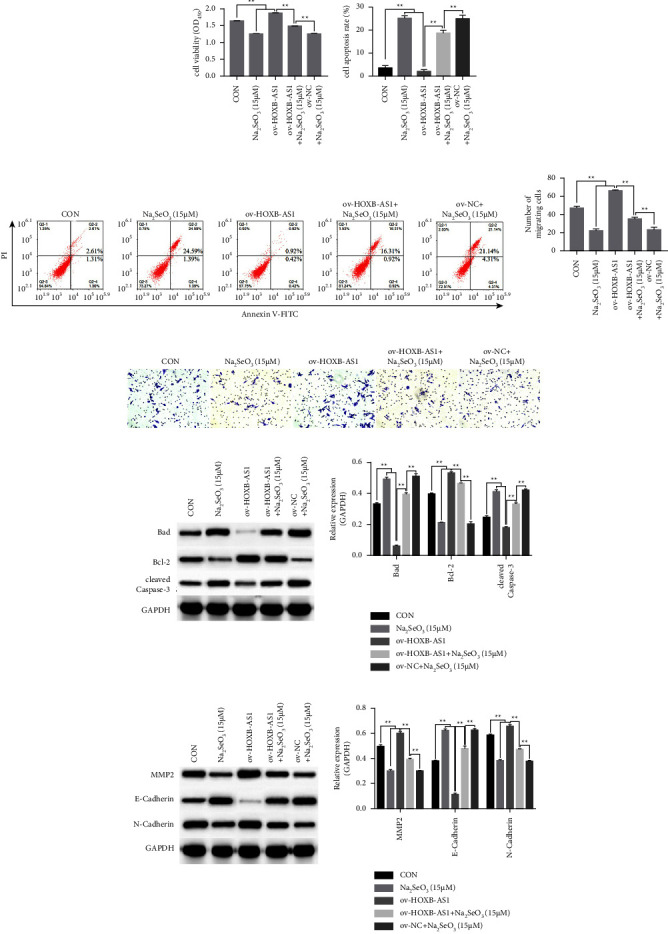
Na_2_SeO_3_ reverses the effect of HOXB-AS1 on proliferation, apoptosis and migration of HGC-27 cells. (a) The viability of HGC-27 cells was determined using a CCK-8 kit. (b, c) The apoptotic ability of HGC-27 cells was assessed using FCM. (d, e) The migration ability of HGC-27 cells was detected by a transwell assay. (f) Relative expression of apoptosis-related proteins detected using western blotting. (g) Relative expression of migration-related proteins, as detected by western blotting. Data are expressed as the mean ± SD (*n* = 3). Data are expressed as the mean ± standard deviation [SD] (*n* = 3), ^*∗∗*^represents *P* < 0.01 and ^*∗*^represents *P* < 0.01.

CCK-8 (Figure 4(a)) and transwell migration (Figures 4(d) and 4(e)) assays revealed that the viability and migration of HGC-27 cells had significantly increased in the ov-HOXB-AS1 group and decreased in the Na_2_SeO_3_ (15 *μ*M) group compared to the CON group (*P* < 0.01). Compared with the ov-HOXB-AS1 + Na_2_SeO_3_ (15 *µ*M) group, viability and migration had significantly increased in the ov-HOXB-AS1 group and decreased in the ov-NC + Na_2_SeO_3_ (15 *μ*M) groups (*P* < 0.01). The opposite trend was observed in the apoptotic ability of HGC-27 cells (Figures 4(b) and 4(c)). HGC-27 cell apoptosis was significantly decreased in the ov-HOXB-AS1 group and increased in the Na_2_SeO_3_ (15 *μ*M) group compared with that in the CON group (*P* < 0.01). Compared to the ov-HOXB-AS1 + Na_2_SeO_3_ (15 *μ*M) group, apoptotic ability was significantly decreased in the OV-HOXB-AS1 group and increased in the ov-NC + Na_2_SeO_3_ (15 *μ*M) group (*P* < 0.01). The protein expression levels of Bad, cleaved caspase-3, and E-cadherin were notably higher in the OV-HOXB-AS1 group and lower in the Na_2_SeO_3_ (15 *μ*M) group than in the CON group (*P* < 0.01). Protein expression was upregulated in the ov-HOXB-AS1 + Na_2_SeO_3_ (15 *μ*M) and ov-NC + Na_2_SeO_3_ (15 *μ*M) groups compared with that in the OV-HOXB-AS1 group (*P* < 0.01). In contrast, the protein expression levels of Bcl-2, MMP2, and N-cadherin were significantly downregulated in the ov-HOXB-AS1 group and upregulated in the Na_2_SeO_3_ (15 *μ*M) group (*P* < 0.01). Similarly, protein expression was downregulated in the ov-HOXB-AS1 + Na_2_SeO_3_ (15 *μ*M) and ov-NC + Na_2_SeO_3_ (15 *μ*M) groups compared with that in the ov-HOXB-AS1 group (*P* < 0.01, Figures 4(f) and 4(g)).

## 4. Discussion

The incidence of gastric cancer has been increasing in recent years, and mortality remains high due to recurrence and metastasis [20]. The incidence of gastric cancer is related to diet, sex, age, region, *Helicobacter pylori* infection status, genetic polymorphisms, and other causes [21]. The rate of early diagnosis is only 10%, meaning that most patients are diagnosed at an advanced stage [22]. Although surgical and perioperative techniques are continuously developing and the safety of gastric cancer resection has greatly improved, various postsurgical complications that seriously affect a patient's quality of life remain issues [23]. At present, most anticancer treatments are nonspecific, killing both tumor and normal cells. Therefore, it is of great clinical significance to explore the pathogenesis of gastric cancer and to develop targeted treatments for its prevention and treatment.

Recent studies have shown that noncoding RNAs (ncRNAs), including ribosomal RNA, transfer RNA, microRNAs, and lncRNAs, are involved in many biological and pathological processes [24, 25]. Cancer is an inherited disease that involves multiple changes in the genome, and differential expression of lncRNAs has been closely related to the occurrence and development of tumors [20]. Seventy-five percent of the human genome is transcribed into RNA, but only a small portion of these transcripts encode proteins. The number of lncRNA genes is very large, and their potential mechanisms of action in cancer largely remain to be explored [26]. For example, increased expression of lncRNA plasmacytoma-variant translocation 1 (PVT1) is associated with an advanced stage and a poor prognosis in patients with ovarian cancer. PVT1 inhibits the expression of miR-214 in ovarian cancer cells by regulating the epithelial-mesenchymal transition process and its interaction with EZH2, which promotes the progression of ovarian cancer [27]. In addition, lncRNAs have been found to play important roles in the development and progression of various cancers [28]. The expression of lncRNA-141 is decreased in gastric cancer cells, and it plays a tumor suppressor role by targeting signal transduction and transcriptional activator 4 [29]; LncRNA-141 is strongly expressed in nonsmall cell lung cancer (NSCLC) cells, where it promotes the proliferation, differentiation, and migration of NSCLC cells by inhibiting the expression of PHLPP1 and PHLPP2 and modulating the PI3K/Akt signaling pathway [30]. HOXBAS1 is located on chromosome 17: 48,543,551–48,551,250. Studies have shown that inhibition of miR-885-3p expression can antagonize HOXB-AS1 knockdown and further affect the expression of HOXB2 in human glioblastoma tissues and cells. The proliferation, migration, and invasion of glioblastoma cells are regulated by HOXB-AS1, which modulates the miR-885-3p/HOXB2 axis [31]. Chen et al. previously showed that the inhibition of HOXB-AS1 expression in multiple myeloma (MM) cells blocks the binding between ELAVL1 and FUT4, thereby regulating the FUT4‐mediated Wnt/*β*-catenin pathway and resulting in decreased proliferation and increased apoptosis of MM cells [32]. Furthermore, HOXB-AS1 plays a key role in several cancers, including glioma and colon cancer, and is presumed to play an oncogenic role in gastric cancer. Moreover, lncRNA HOXB11-AS is overexpressed in gastric cancer and associated with a poor prognosis [33]. Currently, there are no studies on HOXB-AS1 and gastric cancer. Therefore, we hypothesized that HOXB-AS1 is a target for the treatment of gastric cancer. We found that HOXB-AS1 is highly expressed in HGC-27 cells. After HOXB-AS1 silencing, cell proliferation and migration abilities were reduced and the degree of apoptosis increased.

Since the discovery of an inverse relationship between the selenium content of local crops in the United States and the occurrence of cancer in 1969, the anticancer potential of selenium has gradually been discovered [34]. El Bayoumy et al. previously reported several potential actions of selenium in cancer prevention and treatment, including delaying the oxidative damage of DNA, lipids, and proteins; inhibiting the growth of tumor cells; changing the synthesis of DNA, RNA, and proteins; regulating the cell cycle; inducing apoptosis; regulating the expression of p53, COX-2, modified transcription factor-activating protein P, and nuclear factor B [35, 36].

Na_2_SeO_3_ can induce apoptosis in tumor cells undergoing oxidative stress [37], and Gazi et al. found that Na_2_SeO_3_ can inhibit the activation of androgen receptors mediated by interleukin-6 and inhibit the progression of prostate cancer by upregulating the expression of c-Jun [38]. Na_2_SeO_3_ was found to exert antitumor effects by inhibiting tumor angiogenesis in a transplanted canine breast tumor cell model in mice [39]. Na_2_SeO_3_ regulates the IDO1/kynurenine, TLR4, NF- *κ*B, and Bcl2/Bax pathways and attenuates acetic acid-induced colitis in rats [40]. Liu et al. further verified that Na_2_SeO_3_ can increase ROS levels and inhibit the NF-*κ*B signaling pathway, effectively inhibiting the growth, metastasis, and inducing apoptosis of renal cell carcinoma both in vitro and in vivo [41]. This strongly supports the in vivo antitumor effect of Na_2_SeO_3_ and indicates that Na_2_SeO_3_ is a promising therapeutic drug. Although it is often used as an anticancer agent for cancer treatment, the exact mechanism underlying the role of Na_2_SeO_3_ in the development of a therapeutic response remains unclear [42, 43]. In this study, we found that different concentrations of Na_2_SeO_3_ promoted apoptosis in HGC-27 cells and inhibited cell proliferation in a dose-dependent manner. Na_2_SeO_3_ plays an important role in cancer treatment through its important metabolite selenocysteine, a low-molecular-weightselenium-containing amino acid located at the active site of selenocysteine proteins and encoded by the UGA stop codon [44]. It has also been reported that Na_2_SeO_3_ can regulate apoptosis and oxidative damage by reducing the generation of free radicals and inhibiting lipid peroxidation [45, 46]. Currently, there are few reports on the role of lncRNAs in the anticancer potential of Na_2_SeO_3_. We found that Na_2_SeO_3_ inhibited the expression of HOXB-AS1 in gastric cancer cells and regulated the expression of related proteins to inhibit cell proliferation and migration and promote apoptosis after the overexpression of HOXB-AS1 in HGC-27 cells.

In summary, we found that HOXB-AS1 is upregulated in HGC-27, NCI-N87, Kato III, and GES-1 cells. Mechanistic analysis showed that Na_2_SeO_3_ inhibited the proliferation and invasion of gastric cancer cells and enhanced cell apoptosis by downregulating HOXB-AS1 expression. Therefore, the findings of this study highlight the potential therapeutic role of Na_2_SeO_3_ in gastric cancer and suggest that HOXB-AS1 may be a potential therapeutic target. Further in-depth studies are required to confirm these results.

## Data Availability

All data used to support the findings of this study have been included in this article.

## References

[1] Torre L. A., Bray F., Siegel R. L., Ferlay J., Lortet-Tieulent J., Jemal A. (2015). Global cancer statistics, 2012. *CA: A Cancer Journal for Clinicians*.

[2] Waddell T., Verheij M., Allum W., Cunningham D., Cervantes A., Arnold D. (2014). Gastric cancer: ESMO-ESSO-ESTRO clinical practice guidelines for diagnosis, treatment and follow-up. *European Journal of Surgical Oncology*.

[3] Shen L., Shan Y. S., Hu H. M. (2013). Management of gastric cancer in Asia: resource-stratified guidelines. *The Lancet Oncology*.

[4] Chen Y., Lin W. S., Zhu W. F. (2016). Tumor MICA status predicts the efficacy of immunotherapy with cytokine-induced killer cells for patients with gastric cancer. *Immunologic Research*.

[5] Cui Z., Li C., Li X. (2015). Sodium selenite (Na2SeO3) induces apoptosis through the mitochondrial pathway in CNE-2 nasopharyngeal carcinoma cells. *International Journal of Oncology*.

[6] Mazokopakis E. E., Liontiris M. I. (2018). Commentary: health concerns of Brazil nut consumption. *Journal of Alternative & Complementary Medicine*.

[7] Fairweather-Tait S. J., Collings R., Hurst R. (2010). Selenium bioavailability: current knowledge and future research requirements. *The American Journal of Clinical Nutrition*.

[8] Lü J., Zhang J., Jiang C., Deng Y., Özten N., Bosland M. C. (2016). Cancer chemoprevention research with selenium in the post-SELECT era: promises and challenges. *Nutrition and Cancer*.

[9] Bhuyan A. K., Sarma D., Saikia U. K. (2012). Selenium and the thyroid: a close-knit connection. *Indian Journal of Endocrinology and Metabolism*.

[10] Rayman M. P. (2005). Selenium in cancer prevention: a review of the evidence and mechanism of action. *Proceedings of the Nutrition Society*.

[11] Weekley C. M., Harris H. H. (2013). Which form is that? The importance of selenium speciation and metabolism in the prevention and treatment of disease. *Chemical Society Reviews*.

[12] Young R. S., Ponting C. P. (2013). Identification and function of long non-coding RNAs. *Essays in Biochemistry*.

[13] Shi X., Sun M., Liu H., Yao Y., Song Y. (2013). Long non-coding RNAs: a new Frontier in the study of human diseases. *Cancer Letters*.

[14] Gao X., Wen J., Gao P., Zhang G., Zhang G. (2017). Overexpression of the long non-coding RNA, linc-UBC1, is associated with poor prognosis and facilitates cell proliferation, migration, and invasion in colorectal cancer. *OncoTargets and Therapy*.

[15] Yan X., Zhang D., Wu W. (2017). Mesenchymal stem cells promote hepatocarcinogenesis via lncRNA-MUF interaction with ANXA2 and miR-34a. *Cancer Research*.

[16] Yang W., Ning N., Jin X. (2017). The lncRNA H19 promotes cell proliferation by competitively binding to miR-200a and derepressing *β*-catenin expression in colorectal cancer. *BioMed Research International*.

[17] Lammi M. J., Qu C. (2018). Selenium-related transcriptional regulation of gene expression. *International Journal of Molecular Sciences*.

[18] Wang T., Cui Y., Jin J. (2013). Translating mRNAs strongly correlate to proteins in a multivariate manner and their translation ratios are phenotype specific. *Nucleic Acids Research*.

[19] Huang J. Z., Chen M., Chen D. (2017). A peptide encoded by a putative lncRNA HOXB-AS3 suppresses colon cancer growth. *Molecular Cell*.

[20] Feng Y., Jiang J., Hu Z., Yuan J., Zhang T., Pan Y. (2021). Methods for the study of long noncoding RNA in cancer cell signaling. *Methods in Molecular Biology*.

[21] Moradi S. L., Eslami G., Goudarzi H. (2016). Role of *Helicobacter pylori* on cancer of human adipose-derived mesenchymal stem cells and metastasis of tumor cells-an in vitro study. *Tumor Biology*.

[22] Xu W., Liu N. N., Zhu M. P., Sun M. Y. (2019). Progress in prevention and treatment of gastric cancer with traditional Chinese medicine. *World Chinese Journal of Digestology*.

[23] Allum W. H., Griffin S. M., Watson A., Colin-Jones D. (2011). *On Behalf of the Association of Upper Gastrointestinal Surgeons of Great Britain and Ireland, the British Society of Gastroenterology, and the British Association of Surgical Oncology*.

[24] Wapinski O., Chang H. Y. (2011). Long noncoding RNAs and human disease. *Trends in Cell Biology*.

[25] Mattick J. S., Taft R. J., Faulkner G. J. (2010). A global view of genomic information--moving beyond the gene and the master regulator. *Trends in Genetics*.

[26] Djebali S., Davis C. A., Merkel A. (2012). Landscape of transcription in human cells. *Nature*.

[27] Ying C., Hui D., Lewen B., Wenxin L. (2018). LncRNA PVT1 promotes ovarian cancer progression by silencing miR-214. *Cancer Biology & Medicine*.

[28] Liu J. H., Chen G., Dang Y. W., Li C. J., Luo D. Z. (2014). Expression and prognostic significance of lncRNA MALAT1 in pancreatic cancer tissues. *Asian Pacific Journal of Cancer Prevention*.

[29] Zhou X., Xia Y., Su J., Zhang G. (2014). Down-regulation of miR-141 induced by helicobacter pylori promotes the invasion of gastric cancer by targeting STAT4. *Cellular Physiology and Biochemistry*.

[30] Mei Z., He Y., Feng J. (2014). MicroRNA-141 promotes the proliferation of non-small cell lung cancer cells by regulating expression of PHLPP1 and PHLPP2. *FEBS Letters*.

[31] Chen X., Li L. Q., Qiu X., Wu H. (2019). Long non-coding RNA HOXB-AS1 promotes proliferation, migration and invasion of glioblastoma cells via HOXB-AS1/miR-885-3p/HOXB2 axis. *Neoplasma*.

[32] Chen R., Zhang X., Wang C. (2020). LncRNA HOXB-AS1 promotes cell growth in multiple myeloma via FUT4 mRNA stability by ELAVL1. *Journal of Cellular Biochemistry*.

[33] Sun M., Nie F., Wang Y. (2016). LncRNA HOXA11-AS promotes proliferation and invasion of gastric cancer by scaffolding the chromatin modification factors PRC2, LSD1, and DNMT1. *Cancer Research*.

[34] Schrauzer G. N. (2000). Anticarcinogenic effects of selenium. *Cellular and Molecular Life Sciences*.

[35] El-Bayoumy K. (2001). The protective role of selenium on genetic damage and on cancer. *Mutation Research: Fundamental and Molecular Mechanisms of Mutagenesis*.

[36] Reid M. E., Duffield-Lillico A. J., Garland L., Turnbull B. W., Clark L. C., Marshall J. R. (2002). Selenium supplementation and lung cancer incidence: an update of the nutritional prevention of cancer trial. *Cancer Epidemiology, Biomarkers & Prevention*.

[37] Hai-Tao W., Xiang-Liang Y., Zhi-Hong Z., Jing-Ling L. U., Hui-Bi X. U. (2002). EFFECT OF REACTIVE OXYGEN SPECIES ON APOPTOSISINDUCED BY Na_2SeO_3 IN SW480 CELLS. *Acta Biophysica Sinica*.

[38] Gazi M. H., Gong A., Donkena K. V., Young C. Y. (2007). Sodium selenite inhibits interleukin-6-mediated androgen receptor activation in prostate cancer cells via upregulation of c-Jun. *Clinica Chimica Acta*.

[39] Li W., Guo M., Liu Y. (2016). Selenium induces an anti-tumor effect via inhibiting intratumoral angiogenesis in a mouse model of transplanted canine mammary tumor cells. *Biological Trace Element Research*.

[40] Ala M., Jafari R. M., Nematian H., Shadboorestan A., Dehpour A. R. (2022). Sodium selenite modulates Ido1/kynurenine, TLR4, NF-*κ*B and bcl2/bax pathway and mitigates acetic acid-induced colitis in rat. *Cellular Physiology and Biochemistry*.

[41] Liu X., Jiang M., Pang C., Wang J., Hu L. (2022). Sodium selenite inhibits proliferation and metastasis through ROS-mediated NF-*κ*B signaling in renal cell carcinoma. *BMC Cancer*.

[42] Singletary K., Milner J., Milner (2008). Diet, autophagy, and cancer: a review. *Cancer Epidemiology Biomarkers & Prevention*.

[43] Sanmartín C., Plano D., Sharma A. K., Palop J. A. (2012). Selenium compounds, apoptosis and other types of cell death: an overview for cancer therapy. *International Journal of Molecular Sciences*.

[44] Kong F., Hu B., Gao Y. (2015). Fluorescence imaging of selenol in HepG2 cell apoptosis induced by Na2SeO3. *Chemical Communications*.

[45] Ebert R., Ulmer M., Zeck S. (2006). Selenium supplementation restores the antioxidative capacity and prevents cell damage in bone marrow stromal cells in vitro. *Stem Cells*.

[46] Zhang J., Robinson D., Salmon P. (2006). A novel function for selenium in biological system: selenite as a highly effective iron carrier for Chinese hamster ovary cell growth and monoclonal antibody production. *Biotechnology and Bioengineering*.

